# Impact of adjuvant chemotherapy for gliomatosis cerebri

**DOI:** 10.1186/1471-2407-10-424

**Published:** 2010-08-13

**Authors:** Doo-Sik Kong, Sung Tae Kim, Jung-Il Lee, Yeon-Lim Suh, Do Hoon Lim, Won Seog Kim, Ki-Hoon Kwon, Kwan Park, Jong Hyun Kim, Do-Hyun Nam

**Affiliations:** 1 Department of Neurosurgery, Samsung Medical Center, Sungkyunkwan University School of Medicine, Seoul, Korea; 2 Department of Radiology, Center for Imaging Science, Samsung Medical Center, Sungkyunkwan University School of Medicine, Seoul, Korea; 3 Department of Pathology, Samsung Medical Center, Sungkyunkwan University School of Medicine, Seoul, Korea; 4 Department of Radiation Oncology, Samsung Medical Center, Sungkyunkwan University School of Medicine, Seoul, Korea; 5 Division of Hematology-Oncology, Department of Medicine, Samsung Medical Center, Sungkyunkwan University School of Medicine, Seoul, Korea

## Abstract

**Background:**

Gliomatosis cerebri (GC) is characterized by a diffuse infiltration of tumor cells throughout CNS, however, few details are available about the chemotherapeutic effect on GC. The aim of this study was to investigate its clinical course and to determine the efficacy of chemotherapy for GC.

**Methods:**

Between Jan. 1999 and Dec. 2004, 37 GC patients were diagnosed by biopsy and treated with radiotherapy in a single institution. To determine the efficacy of chemotherapy for GC, we retrospectively reviewed their clinical courses. The study cohort was divided into 2 groups, those with and without receiving post-radiotherapy adjuvant chemotherapy such as temozolomide or nitrosourea-based chemotherapy.

**Results:**

Nineteen patients with adjuvant chemotherapy were assigned to the chemotreatment group and 18 with radiotherapy alone were assigned to the control group. Mean survival for chemotreatment group and control group were 24.2 and 13.1 months, respectively (p = 0.045). Time to progression for these groups were 16.0 and 6.0 months, respectively (p = 0.007). Overall review of the clinical course of patients with GC provided that early appearance of new contrast-enhancing lesions within 6 months from the initial diagnosis and higher histological grade were closely associated with poor survival (p < 0.001 and p = 0.008).

**Conclusion:**

Adjuvant chemotherapy following radiotherapy could prolong the survival in patients with GC. In addition, newly developed contrast-enhanced lesions on the follow-up MR images indicate the progression of GC.

## Background

Gliomatosis cerebri (GC) is a rare variant of glioma. It is characterized by a diffuse infiltration of malignant glial cells throughout large regions of the central nervous system [1-6] and a relative preservation of the neural architecture. Since Nevin first used the term gliomatosis cerebri in 1938, describing the histological finding of glial overgrowth of the brain [7], the World Health Organization now defines GC as a diffuse glial tumor that extensively infiltrates the brain and involves more than multiple lobes (frequently bilaterally) [8]. Jennings et al. reported that GC can be divided into two forms based on descriptive neuropathological grounds [9,10]. Type I GC consists of well-differentiated low-grade gliomas without contrast enhancement and its clinical course is often mild, with slow progression. Once the tumor begins to progress and cancerous dedifferentiation is observed, this type of tumor is referred to as type II GC, leading to clinical deterioration.

In the treatment of GC, radiotherapeutic effects as the first-line treatment have been supported by several studies [3,6,7,11]. However, chemotherapeutic effects as an adjuvant treatment or first-line treatment are not definitely proved yet [10,12,13]. Here, we present a series of 37 GC patients who underwent radiotherapy for GC. To identify the efficacy of adjuvant chemotherapy for GC, we analyzed and compared clinical outcome between adjuvant chemotherapy group and radiotherapy alone group.

## Methods

### Including criteria

We retrospectively identified 45 consecutive patients diagnosed with GC who were examined at a single institution between Jan. 1999 and Dec. 2004. Of these, 37 patients were included in this study based on the following criteria: 1) MRI evidence of diffuse, increased signal intensity on the T2-weighted or FLAIR images accompanied by a low or absent signal in the affected areas on T1 images, 2) lesions involving more than two cerebral lobes, 3) available serial MR images and clinical follow-up data, 4) who underwent radiotherapy for GC. Eight patients were excluded because of unavailable follow-up data or no treatment history. To rule out other inflammatory diseases, 24 patients underwent stereotactic biopsy and 13 patients did partial tumor resection immediately after radiological diagnosis. Information about treatment response was obtained by reviewing the patients' radiological data and clinical medical records. Immediately after surgery, local radiotherapy was performed in all patients. The extent of the radiotherapy was determined by the area of tumor involvement. A median 5800 cGy (5400-6000 cGy) were delivered according to routine treatment plans. **The study was approved by the local ethic committee**.

### Patients characteristics

The chemotreatment group received adjuvant TMZ or nitrosourea-based chemotherapy following radiotherapy (n = 19), and the control group underwent radiotherapy alone (n = 18). There were no statistically significant differences in baseline characteristics between the 2 groups (Table 1). For this cohort group, twenty-two patients were men (59.5%) and 15 patients were women (40.5%). The mean age at presentation was 41.2 years (range: 11-67 years). The median KPS at the time of initial diagnosis was 80 (range: 70-100). The MR images demonstrated a variety of findings including presence of: a dominant mass formation (18 cases) or a diffuse infiltration pattern (19 cases), initial dimly enhanced lesions or not (6 vs. 31 cases), brainstem involvement or not (11 vs. 26 cases), and tumor extent, i.e., involving two lobes or ≥ 3 lobes (13 vs. 24 cases). Based on the histopathological data, 23 patients were diagnosed as having a tumor with low-grade features (grade II) and 14 patients were diagnosed as having tumor with high-grade features (grade III).

**Table 1:** Characteristics of the patients with gliomatosis cerebri

Variable	Chemotreatment group (n = 19)	Control group (n = 18)	P
Age			0.579
Median	44.4	43.5	
range	13-58	11-67	
Gender			0.638
Male	12	10	
Female	7	8	
KPS score			0.713
≥ 70	16	15	
< 70	3	3	
Grade			0.420
Low	13	10	
High	6	8	
Mass formation			0.738
Dominant mass	9	9	
Diffuse infiltration	10	9	
Brainstem involvement			0.717
Yes	6	5	
No	13	13	
Hemisphere involvement			0.654
Unilateral	11	11	
Bilateral	8	7	
Operation			0.823
Biopsy	12	12	
Partial tumor removal	7	6	

### Adjuvant chemotherapy

19 of 37 patients (51.4%) received adjuvant chemotherapy after radiotherapy, while 18 other patients underwent local radiotherapy alone. Chemotherapeutic regimens included temozolomide (TMZ) (200 mg/m^2 ^× consecutive 5 days per month, 3-9 cycles) and nitrosourea-based chemotherapy such as BCNU (carmustine-[1,3-bis (2-chloroethyl)-1-nitrosourea]), PCV (procarbazine/lomustine/vincristine). The choice of chemotherapeutic regimens was dependent upon how much tumors had oligodendroglial components. At an earlier period, PCV chemotherapy was given for 5 patients with tumor which dominantly contained oligodendroglial components, while BCNU treatment (4 patients) was performed for tumors with dominant astrocytic components. However, since TMZ treatment had been widely introduced into our institution in 2002, 10 patients received TMZ treatment regardless of the cellular components.

All patients were assessed by serial MR images with contrast enhancement. The follow-up images were evaluated for progression versus no progression by a radiologist (Kim ST) blinded to the pathological and clinical findings. During the follow-up period, we sometimes observed new contrast-enhancing lesions, which had been absent in the previous studies. These contrast-enhancing lesions were patched or punctuated lesions[14,15] and located in the mid-portion of widely infiltrated lesions on FLAIR images. They could be differentiated from radiation necrosis by several MR sequences (diffusion/perfusion weighted images, MR spectroscopy, or FDG-PET) [16-20]. It was difficult to apply the tumor response criteria commonly used for high-grade gliomas because of the lack of discrete and measurable tumor margins. Tumor progression was assessed by semi-quantitative analysis of the tumor extent in T2-weighted or FLAIR-weighted MR images using commercially available image analysis software (SCION Corp.).

In cases of tumor recurrence or progression, we performed surgical resection of the enhancing lesions in 10 patients or gamma knife radiosurgery in 6 patients. All patients with tumor recurrence or progression received 2^nd^-line chemotreatment.

### Statistical analysis

Overall survival and progression-free survival were calculated using the Kaplan-Meier method and groups were compared with the log-rank test. For statistical analysis, age, gender, KPS, histological grade, degree of resection, and involvement of brainstem were compared using Fisher exact test or Mann-Whitney test between the chemotreatment group and the control group. The Cox proportional hazards model with a stepwise procedure was used in the multi-variate survival analysis to assess the prognostic factors for survival. P values of less than 0.05 were considered statistically significant for all tests, using the SPSS software (SPSS version 10.0, Chicago, IL).

## Results

**Figure 1:**
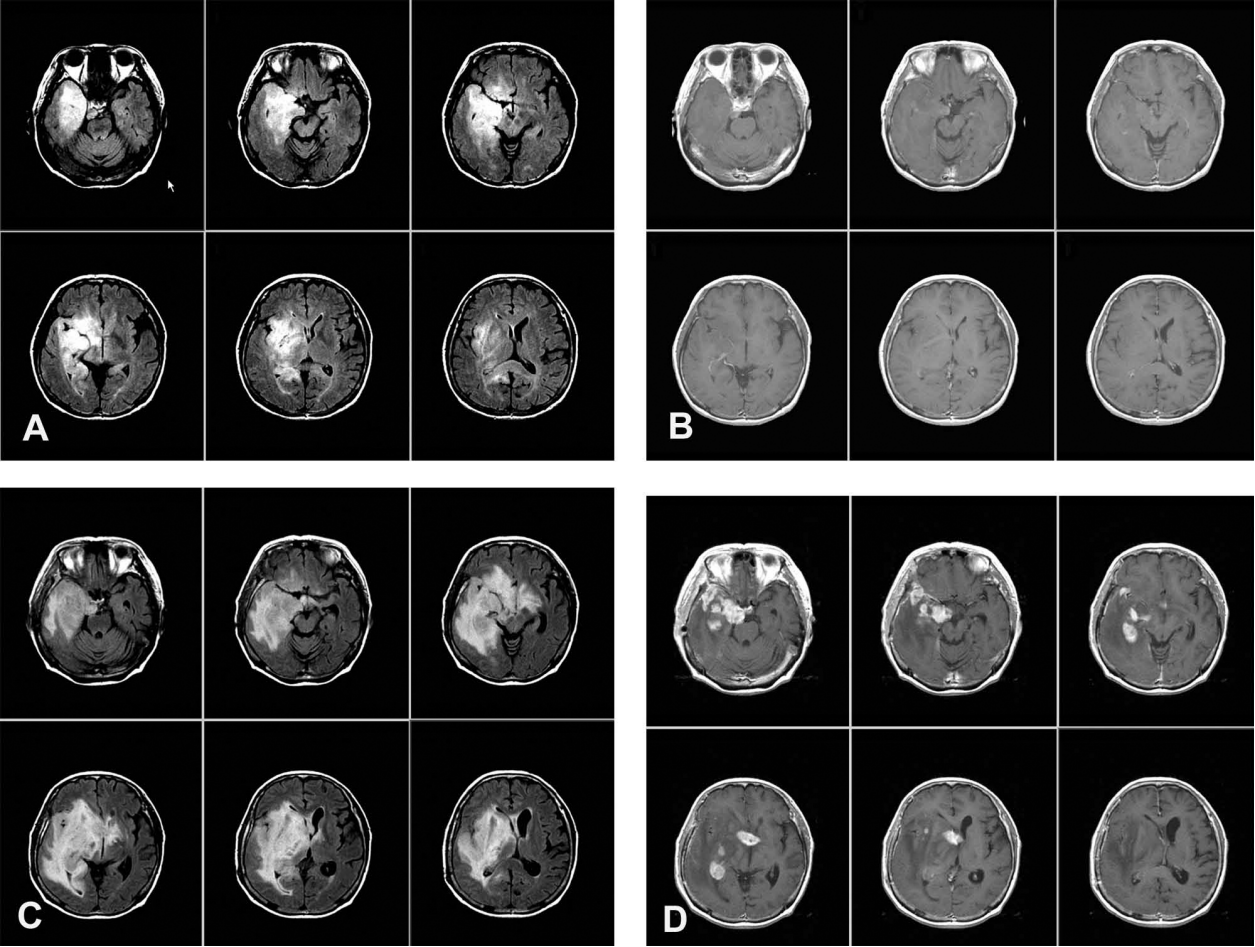
**Serial MR images of gliomatosis cerebri**. Case 2: (A & B) at the initial presentation, highly infiltrated lesion is found on the FLAIR image, but there is no enhancing lesion on the T1 weighted enhanced image (T1WI), (C & D) After 3 months, the tumor is widely spread on the FLAIR images and new contrast-enhancing lesions diffusely appears on the T1WI.

During the follow-up period, median time to new appearance of contrast-enhancing lesions was 12.0 months (95% CI: 7.7-16.3 months). In particular, early appearance of new contrast enhancing lesions within 6 months was found in 11 of the 37 patients (29.7%). In most cases, these patched or punctuated lesions were developed in the small area of overall tumor involvement at the initial stage and were strongly enhanced with the contrast dye (Fig. 1). Overall review of clinical course demonstrated that early appearance of new contrast enhancing lesions within 6 months was closely related to poor survival (p < 0.001 by log-rank test). Furthermore, early appearance of new enhancing lesions within 6 months was also a significant independent variable for tumor progression (p < 0.001 by log-rank test).

### Treatment outcome

**Figure 2:**
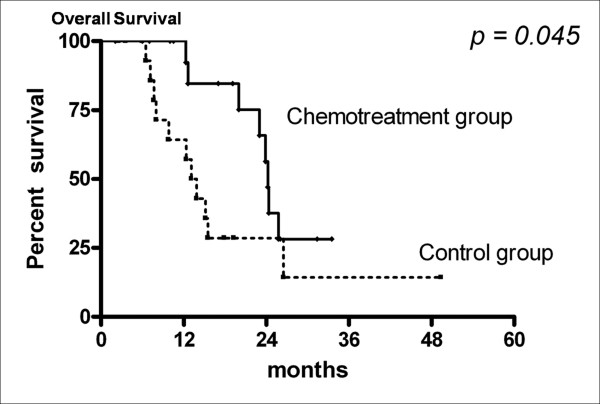
**Comparison of overall survival between patients treated with adjuvant chemotherapy plus radiotherapy (chemotreatment group) and those with radiotherapy alone (control group)**.

Twenty-five patients were still alive at the time of this analysis and the median follow-up period was 12.6 months (range: 7.1-49.4 months). Median overall survival in the chemotreatment group was 24.2 months (95% CI: 23.4-24.9 months), compared with 13.1 months (95% CI: 10.4-15.9 months) in the control group. Kaplan-Meier analysis showed that median overall survival in the chemotreatment group was longer than that in the control group (p = 0.045 by log-rank test, Fig. 2). Age, gender, KPS, cellular components, degree of resection and brainstem involvement were not identified as independent prognostic variables. For Cox proportional hazard model, no chemotherapy (HR 6.385, 95% CI: 1.8-22.6, p = 0.004) was strongly associated with poor survival even after adjustment for age, sex, KPS, cellular components, and brainstem involvement. Higher histological grade (HR 4.434, 95% CI: 1.312-14.982, p = 0.017) was also associated with dismal prognosis (table 2). Between patients treated with TMZ and those with nitrosourea-based chemotreatment, there was no significant difference of survival (p > 0.05 by log-rank test).

**Table 2:** Cohort of patients with Gliomatosis Cerebri: Prognostic variables for survival

Independent variables	Univariate analysis	Multivariable analysis	Hazard ratio(R)	95% CI for Hazard ratio	
				
				Lower	Upper
Age (≤ 55 years)	*P *= 0.523		1.212	0.886	3.178
Sex	*P *= 0.872		1.082	0.395	2.886
KPS(≥ 70)	*P *= 0.454		0.825	0.564	4.867
Cellular component	*P *= 0.624		0.531	0.348	0.809
Histological grade	*P = *0.023	*P = *0.017	4.434	1.312	14.982
Adjuvant chemotherapy	*P = *0.045	*P = *0.004	6.385	1.885	22.685
Brainstem involvement	*P = *0.952		2.312	0.285	13.524

**Figure 3:**
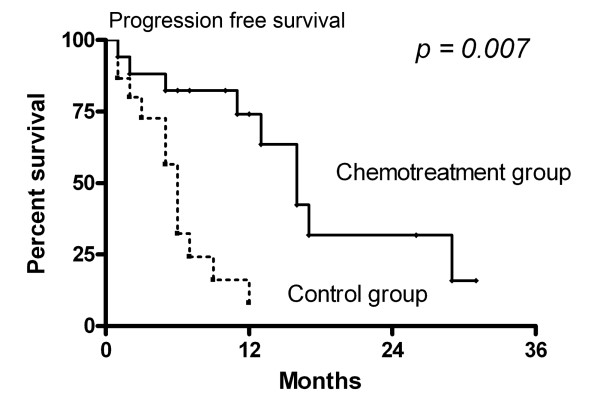
**Comparison of progression-free survival between patients treated with adjuvant chemotherapy plus radiotherapy (chemotreatment group) and those with radiotherapy alone (control group)**.

Median progression-free survival in the chemotreatment group was 16.0 months (95% CI: 11.7-20.2 months), compared with 6.0 months (95% CI: 4.9-7.1 months) in the control group. Adjuvant chemotherapy (OR 8.250, 95% CI: 1.8-38.7, p = 0.007 by log-rank test, Fig. 3) was a significant independent factor for progression after adjustment of age, sex, KPS, cellular components, and histological grade.

## Discussion

### Progression of GC: significance of the newly developed contrast-enhanced lesion

GC is an infrequent and controversial diagnosis, because the definition and pathophysiology have not been accurately defined yet. Recent studies using antemortem diagnostic criteria by MRI have revealed that the prognosis for GC is generally poor and the overall survival relatively short [1,2,6,8,9,21-27]. In the respective of radiological view, 19 of 38 patients in this study could be classified as de novo (primary) GC, whereas the others resulted from the spreading of a focal glioma (secondary GC) [10]. Vates et al. suggested that GC begins as either a low- or high-grade neoplasm without a focal mass, which progresses to form a focal mass similar to the natural history of low grade glioma [11]. However, GC should not be diagnosed by histopathological finding, but by radiographic findings. Furthermore, in most cases, difficulties in interpreting histological specimen are attributable to scattering of tumor cells and small amount of tissue specimens obtained by stereotactic biopsy. Therefore, it was too difficult to clarify the specific histological types (astrocyte-dominant or oligodendroglial-dominant) based on the tissue specimen. In this study, an interval between early stage and tumor progression in GC was extremely variable in each other.

We speculated that this change of enhancement pattern might imply a malignant progression of GC. In the current study, we found that early appearance of new patched or punctuated enhancing lesions was closely correlated with the poor survival and tumor progression. As a result, these findings supported that malignant transformation of GC is similar to that of low-grade gliomas. Tumor progression of GC used to be more rapid than that of low-grade gliomas, which can be explained by the following hypotheses. GC may contain highly invasive stem-like tumor cells and therefore shows a tendency to involve more lobes and progress rapidly [28]. Otherwise, it can be interpreted as a transformation from angiogenesis-independent growth to an angiogenesis-dependent phenotype driven by stem-like cells [29]. In the future, a detailed review of the clinical course in GC will contribute to our understanding of malignant progression of tumors [30].

### Therapeutic effects of adjuvant chemotherapy for GC

To date, little is known about the therapeutic effect of adjuvant chemotherapy for GC. However, some researchers demonstrated that upfront TMZ treatment was effective for patients with GC [10,12,13]. By using a retrospective case-cohort study, we investigated the efficacy of adjuvant chemotherapy such as TMZ or nitrosourea-based chemotherapy for GC. In this study, we demonstrated that adjuvant chemotherapy following radiotherapy was effective for the prolongation of survival and delay of tumor progression. This suggests that adjuvant chemotherapy as well as first-line chemotherapy also provide improvement of survival in patients with GC. Furthermore, multivariate analysis revealed that GC patients with higher histological grade had shorter survival and earlier tumor progression than those with lower histological grade. Therefore, during the follow-up period, higher histological grade at the initial presentation or earlier appearance of enhancement should warrant dismal prognosis.

However, it is not certain that adjuvant chemotherapy should be required following radiotherapy for GC, because this retrospective study has some drawbacks. Even though these two groups were relatively balanced with respect to known prognostic variables, they were not randomly-allocated groups, but were groups determined by physician's preference. We should recognize that these selection biases could account for its effect on survival. Moreover, it was another important limitation in this study that chemotreatment group was composed of heterogeneous populations, for example, those treated with TMZ or nitrosourea-based chemotherapy.

## Conclusion

In conclusion, adjuvant chemotherapy following radiotherapy could prolong the survival in patients with GC. In addition, newly developed contrast-enhanced lesions on the follow-up MR images indicate the progression of GC.

## Competing interests

The authors declare that they have no competing interests.

## Authors' contributions

DN was responsible for the conception and design of the study; DK and SK performed the collection and assembly of data; DK, SK, JL, KK, KP, and JK performed the statistical analysis and interpretation of data; DK, SK and YS participated in writing the manuscript; DL, WK, and DN revised the manuscript. All authors read and approved the final manuscript.

## Pre-publication history

The pre-publication history for this paper can be accessed here:

http://www.biomedcentral.com/1471-2407/10/424/prepub

## References

[B1] Couch JR Weiss SA Gliomatosis cerebri. Report of four cases and review of the literature Neurology 1974 24 6 504 511 4499966 10.1212/wnl.24.6.504

[B2] del Carpio-O'Donovan R Korah I Salazar A Melancon D Gliomatosis cerebri Radiology 1996 198 3 831 835 8628879 10.1148/radiology.198.3.8628879

[B3] Artigas J Cervos-Navarro J Iglesias JR Ebhardt G Gliomatosis cerebri: clinical and histological findings Clin Neuropathol 1985 4 4 135 148 4053456

[B4] Choi D Schulz U Seex K Gliomatosis cerebri: a brain tumour which is too difficult to treat? Scott Med J 1998 43 3 84 86 9682295 10.1177/003693309804300308

[B5] Mawrin C Kirches E Schneider-Stock R Scherlach C Vorwerk C Von Deimling A Van Landeghem F Meyermann R Bornemann A Muller A Romeike B StoltenburgDidinger G Wickboldt J Pilz P Dietzmann K Analysis of TP53 and PTEN in gliomatosis cerebri Acta Neuropathol (Berl) 2003 105 6 529 536 10.1007/s00401-003-0674-5 12734658

[B6] Kim DG Yang HJ Park IA Chi JG Jung HW Han DH Choi KS Cho BK Gliomatosis cerebri: clinical features, treatment, and prognosis Acta Neurochir (Wien) 1998 140 8 755 762 10.1007/s007010050176 9810441

[B7] Perkins GH Schomer DF Fuller GN Allen PK Maor MH Gliomatosis cerebri: improved outcome with radiotherapy Int J Radiat Oncol Biol Phys 2003 56 4 1137 1146 10.1016/S0360-3016(03)00293-1 12829152

[B8] Louis DN Ohgaki H Wiestler OD Cavenee WK Burger PC Jouvet A Scheithauer BW Kleihues P The 2007 WHO classification of tumors of the central nervous system Acta Neuropathol 2007 114 2 97 109 10.1007/s00401-007-0243-4 17618441 PMC1929165

[B9] Jennings MT Frenchman M Shehab T Johnson MD Creasy J LaPorte K Dettbarn WD Gliomatosis cerebri presenting as intractable epilepsy during early childhood J Child Neurol 1995 10 1 37 45 10.1177/088307389501000111 7539465

[B10] Sanson M Cartalat-Carel S Taillibert S Napolitano M Djafari L Cougnard J Gervais H Laigle F Carpentier A Mokhtari K Taillandier L Chinot O Duffau H Honnorat J Hoang-Xuan K Delattre JY Initial chemotherapy in gliomatosis cerebri Neurology 2004 63 2 270 275 15277619 10.1212/01.wnl.0000129985.39973.e4

[B11] Vates GE Chang S Lamborn KR Prados M Berger MS Gliomatosis cerebri: a review of 22 cases Neurosurgery 2003 53 2 261 271 discussion 271 10.1227/01.NEU.0000073527.20655.E6 12925240

[B12] Sanson M Napolitano M Cartalat-Carel S Taillibert S Gliomatosis cerebri Rev Neurol (Paris) 2005 161 2 173 181 15798516 10.1016/s0035-3787(05)85020-9

[B13] Kaloshi G Everhard S Laigle-Donadey F Marie Y Navarro S Mokhtari K Idbaih A Ducray F Thillet J Hoang-Xuan K Delattre JY Sanson M Genetic markers predictive of chemosensitivity and outcome in gliomatosis cerebri Neurology 2008 70 8 590 595 10.1212/01.wnl.0000299896.65604.ae 18285534

[B14] Chaichana KL McGirt MJ Laterra J Olivi A Quinones-Hinojosa A Recurrence and malignant degeneration after resection of adult hemispheric low-grade gliomas J Neurosurg 2010 112 1 10 7 10.3171/2008.10.JNS08608 19361270

[B15] Chaichana KL McGirt MJ Niranjan A Olivi A Burger PC Quinones-Hinojosa A Prognostic significance of contrast-enhancing low-grade gliomas in adults and a review of the literature Neurol Res 2009 31 9 931 9 10.1179/174313209X395454 19215664

[B16] Cha S Johnson G Wadghiri YZ Jin O Babb J Zagzag D Turnbull DH Dynamic, contrast-enhanced perfusion MRI in mouse gliomas: correlation with histopathology Magn Reson Med 2003 49 5 848 855 10.1002/mrm.10446 12704767

[B17] Law M Hamburger M Johnson G Inglese M Londono A Golfinos J Zagzag D Knopp EA Differentiating surgical from non-surgical lesions using perfusion MR imaging and proton MR spectroscopic imaging Technol Cancer Res Treat 2004 3 6 557 565 15560713 10.1177/153303460400300605

[B18] Nagesh V Chenevert TL Tsien CI Ross BD Lawrence TS Junck L Cao Y Quantitative characterization of hemodynamic properties and vasculature dysfunction of high-grade gliomas NMR Biomed 2007 20 6 566 577 10.1002/nbm.1118 17221937

[B19] Pivawer G Law M Zagzag D Perfusion MR imaging and proton MR spectroscopic imaging in differentiating necrotizing cerebritis from glioblastoma multiforme Magn Reson Imaging 2007 25 2 238 243 10.1016/j.mri.2006.09.028 17275620 PMC1847362

[B20] Yang S Law M Zagzag D Wu HH Cha S Golfinos JG Knopp EA Johnson G Dynamic contrast-enhanced perfusion MR imaging measurements of endothelial permeability: differentiation between atypical and typical meningiomas AJNR Am J Neuroradiol 2003 24 8 1554 1559 13679270 PMC7974003

[B21] Shin YM Chang KH Han MH Myung NH Chi JG Cha SH Han MC Gliomatosis cerebri: comparison of MR and CT features AJR Am J Roentgenol 1993 161 4 859 862 8372774 10.2214/ajr.161.4.8372774

[B22] Pyhtinen J Proton MR spectroscopy in gliomatosis cerebri Neuroradiology 2000 42 8 612 615 10.1007/s002340000343 10997568

[B23] Pyhtinen J Paakko E A difficult diagnosis of gliomatosis cerebri Neuroradiology 1996 38 5 444 448 10.1007/BF00607270 8837088

[B24] Spagnoli MV Grossman RI Packer RJ Hackney DB Goldberg HI Zimmerman RA Bilaniuk LT Magnetic resonance imaging determination of gliomatosis cerebri Neuroradiology 1987 29 1 15 18 10.1007/BF00341031 3822096

[B25] Peretti-Viton P Brunel H Chinot O Daniel C Barrie M Bouvier C Figarella-Branger D Fuentes S Dufour H Grisoli F Histological and MR correlations in Gliomatosis cerebri J Neurooncol 2002 59 3 249 259 10.1023/A:1019934901750 12241123

[B26] Keene DL Jimenez C Hsu E MRI diagnosis of gliomatosis cerebri Pediatr Neurol 1999 20 2 148 151 10.1016/S0887-8994(98)00132-5 10082346

[B27] Essig M Schlemmer HP Tronnier V Hawighorst H Wirtz R van Kaick G Fluid-attenuated inversion-recovery MR imaging of gliomatosis cerebri Eur Radiol 2001 11 2 303 308 10.1007/s003300000587 11218032

[B28] Sanai N Alvarez-Buylla A Berger MS Neural stem cells and the origin of gliomas N Engl J Med 2005 353 8 811 822 10.1056/NEJMra043666 16120861

[B29] Sakariassen PO Prestegarden L Wang J Skaftnesmo KO Mahesparan R Molthoff C Sminia P Sundlisaeter E Misra A Tysnes BB Chekenya M Peters H Lende G Kalland KH Øyan AM Petersen K Jonassen I van der Kogel A Feuerstein BG Terzis AJ Bjerkvig R Enger PØ Angiogenesis-independent tumor growth mediated by stem-like cancer cells Proc Natl Acad Sci USA 2006 103 44 16466 16471 10.1073/pnas.0607668103 17056721 PMC1618812

[B30] Kong DS Kim MH Park WY Suh YL Lee JI Park K Kim JH Nam DH The progression of gliomas is associated with cancer stem cell phenotype Oncol Rep 2008 19 3 639 643 18288395

